# Supplementary material for: Identifying the key steps determining the selectivity of toluene methylation with methanol over HZSM-5
Source: Nat Commun. 2021 Jun 17;12:3725. doi: 10.1038/s41467-021-24098-5 (PMC8211704; doi:10.1038/s41467-021-24098-5)
Supplement: Supplementary file 1 — Supplementary Information [file 41467_2021_24098_MOESM1_ESM.pdf]

Supplementary Information

Identifying the Key Steps Determining the Selectivity of Toluene Methylation with Methanol over HZSM-5

Qingteng Chen[1], Jian Liu[1,*] and Bo Yang[1,*]

[1]*School of Physical Science and Technology, ShanghaiTech University, 393 Middle Huaxia Road, Shanghai 201210, China*

*E-mail: liujian@shanghaitech.edu.cn; yangbo1@shanghaitech.edu.cn

Table of Contents

Supplementary Note 1. Stepwise mechanism results..3

Supplementary Note 2. NPT optimization of lattice parameters 5

Supplementary Note 3. Uncertainty analysis on MTD simulation results 6

Supplementary Note 4. Free energy of proton-MX/OX in the MTD-PX simulation 8

Supplementary Note 5. 2D free energy surface of MTD-MX..................................9

Supplementary Note 6. Structural analysis of the MTD simulations 10

Supplementary Note 7. Collective variables and quadratic walls.........................13

Supplementary Note 8. Convergence tests of slow-growth simulations................14

Supplementary Note 9. Diffusion barriers..16

Supplementary Note 10. Trajectories of CV1-CV2 in MTD-PX/MX 17

Supplementary Note 1. Stepwise mechanism results

According to the stepwise mechanism as shown in Supplementary Figure 1, the methylation reaction was divided into two parts, i.e. adsorbed methanol dissociation to form surface methyl groups and surface methyl groups reacting with toluene to form proton-xylene.

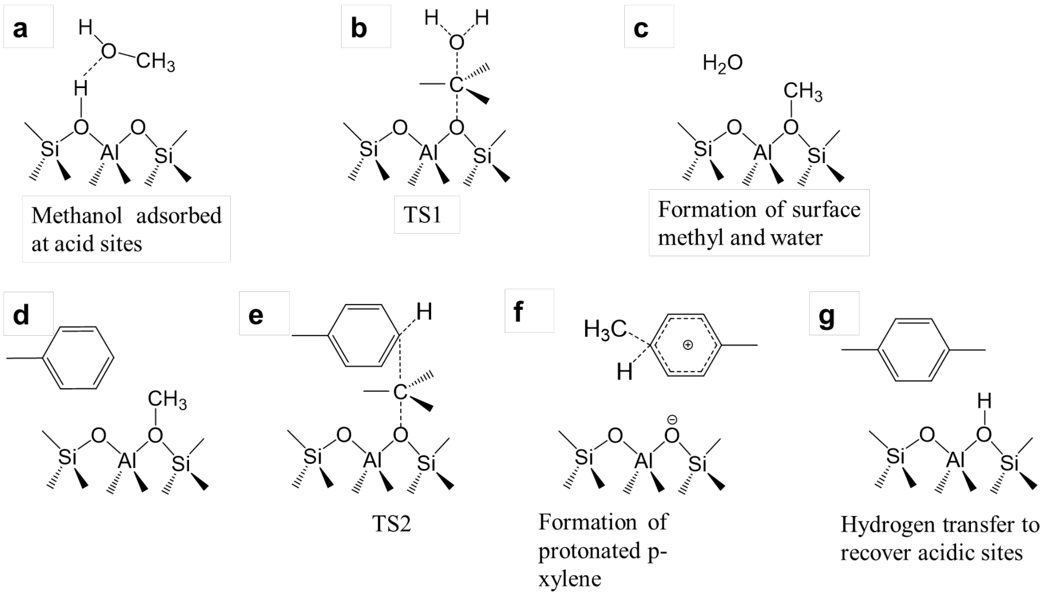

Supplementary Figure 1. Stepwise mechanism of toluene methylation reaction.

MTD method was used to study these reactions. As for the surface methyl formation reaction, CV1 was the CN of O_{me}–C_{me}, CV2 was the CN of C_{me}–O_{Z}, *nn*, *nd*, r_0 were 5, 10, 1.5 Å respectively (according to Equation 1 in the main text). The initial height of Gaussian hill was 5.251 kJ mol^{-1}. All other parameters are the same with the settings in the main text (details shown in Methods section). Supplementary Figure 2 shows that the free energy barrier is greater than 150 kJ mol^{-1}, which is close to the value of 152 kJ mol^{-1} reported in the literature.[1] One can find that the free energy barrier of surface methyl group formation is much higher than the effective barrier of the concerted mechanism, and therefore the stepwise mechanism should be preferred under the conditions considered in our work.

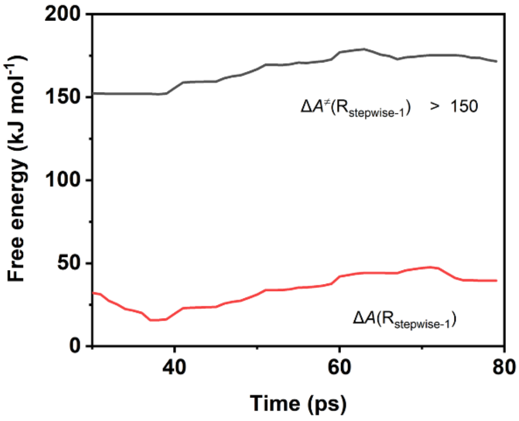

Supplementary Figure 2. The curve showing free energy barrier and reaction free energy of surface methyl groups formation against time.

As for the proton-PX/MX formation reaction, CV1 was the CN of C_{me}–O_z, *nn*, *nd*, r_0 were 6, 12 and 1.5 Å, respectively; CV2 was the CN of C_{me}–$C_{p/m}$, *nn*, *nd*, r_0 were 6, 12 and 2.0 Å, respectively. Other parameters remain unchanged. Supplementary Figure 3 shows the free energy barriers are quite similar and proton–PX is more stable than proton–MX in HZSM-5, which are consistent with the results of direct methylation mechanism.

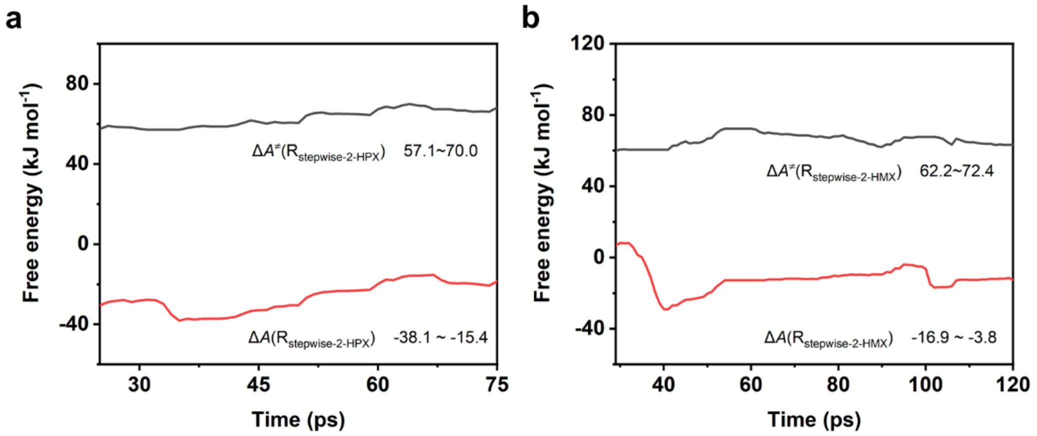

Supplementary Figure 3. The curve of free energy barrier and reaction free energy of (a) proton–PX and (b) proton–MX formation reactions against time.

Supplementary Note 2. NPT optimization of lattice parameters

A 50 ps NPT simulation was performed at the temperature of 670 K and the pressure of 1 bar, to confirm the reliability of lattice parameters used in the current work. By averaging over the range from 5 to 50 ps (the first 5 ps was used to equilibrate the system), the optimized lattice constants were evaluated to be a = 20.224 Å, b = 20.014 Å, c = 13.485 Å, which is very close to the values used in the original manuscript with a negligible discrepancy of ~ 0.02 Å (~ 0.1 %).

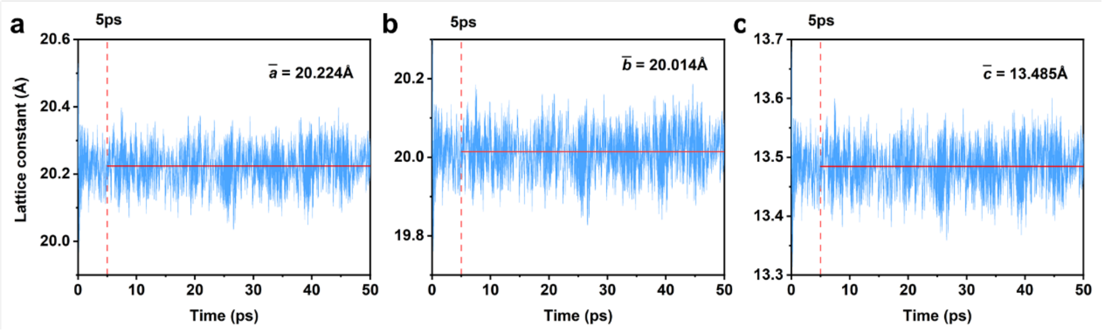

Supplementary Figure 4. Trajectories of the lattice parameters *a*, *b* and *c* in the NPT simulation.

Supplementary Note 3. Uncertainty analysis on MTD simulation results

Based on the method proposed by G. Bussi et al.,[2] we obtained the average 1D free energy profile after t_{fill} that all the free energy minima are filled with Gaussian hills, then computed the values and uncertainties of the free energy barrier and reaction free energy. According to the free energy barrier and reaction free energy curves shown in Supplementary Figure 5, we determined the t_{fill} as 60 ps for the MTD-PX simulation and 80 ps for the MTD-MX simulation. Block average method was used here with 4 ps in each block to estimate the uncertainties (standard deviation) of the free energy barrier and reaction free energy. All these results are shown in Supplementary Figure 6 and the error interval is acceptable. The IS was used as the reference point to confirm the uncertainties of the methylation barrier and reaction energy; the FS was used as the reference point to calculate the error of the isomerization barrier.

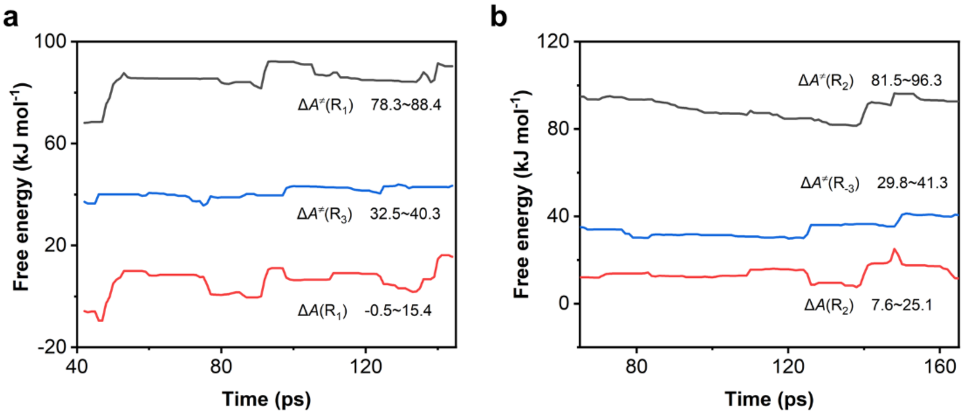

Supplementary Figure 5. Methylation barrier, isomerization barrier and reaction free energy against time in (a) MTD-PX and (b) MTD-MX simulations.

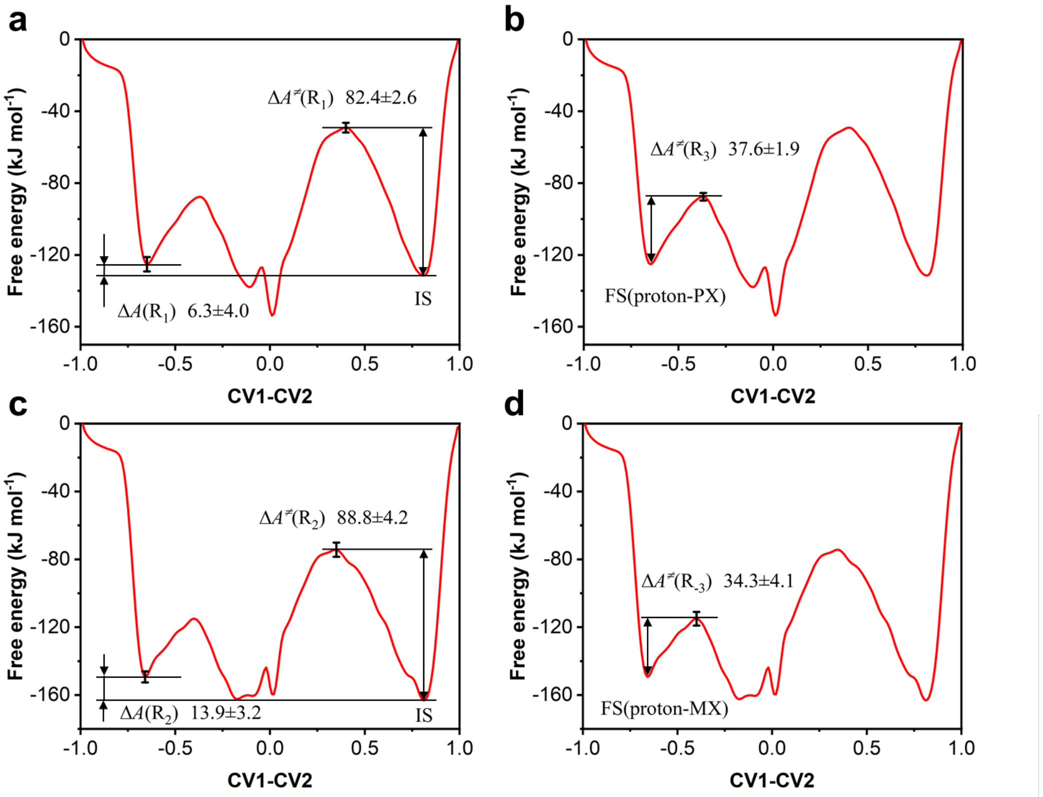

Supplementary Figure 6. Average 1D free energy profiles with error bars (standard deviation) of (a) MTD-PX (IS as reference point); (b) MTD-PX (proton-PX as reference point); (c) MTD-MX (IS as reference point); (d) MTD-MX (proton-MX as reference point) simulations.

Supplementary Note 4. Free energy of proton-MX/OX in the MTD-PX simulation

Supplementary Figure 7 shows the free energy change curve of proton-MX/OX against time in MTD-PX. The free energy of proton-MX/OX remains the same or suddenly decreases during simulation, suggesting that these regions are very easy to overfill and the corresponding free energies are not reliable.

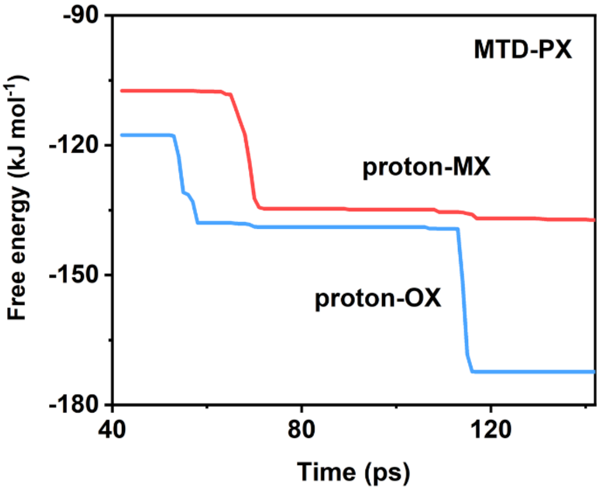

Supplementary Figure 7. The free energy change curve of proton-MX/OX against time in MTD-PX simulation.

Supplementary Note 5. 2D free energy surface of MTD-MX

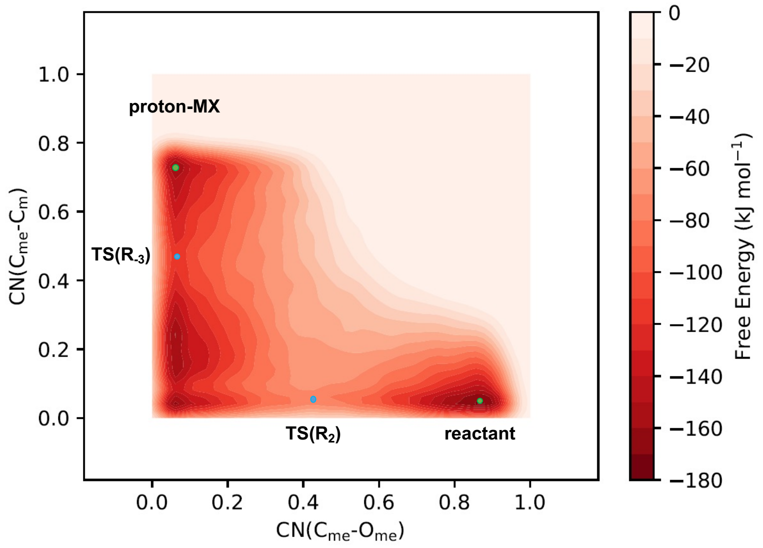

Supplementary Figure 8. Two-dimensional free energy surface of the methylation of toluene obtained from MTD-MX simulation.

Supplementary Note 6. Structural analysis of the MTD simulations

In order to analyze the structure of some metastable states and transition states in 2D free energy surface of MTD-PX/MX, we set up some criteria to select the structures for further analysis.

Taking MTD-PX as an example, the approximate areas of the IS, TS(R_1), TS(R_3) and FS (proton-PX) states were obtained in the 2D free energy surface using a certain energy range. The energy range for the IS and FS region is from $A_{IS/FS}$ to $A_{IS/FS}$ + k_BT, while for TS this is from A_{TS} - 0.5 k_BT to A_{TS} + 0.5 k_BT (1 k_BT = 5 kJ mol^{-1} at 670 K). Then rectangle regions that meet the criteria were selected, as shown in Supplementary Table 1. All the frames that within these rectangle regions were analyzed, and the structural analysis was performed accordingly.

Supplementary Table 1. The rectangle regions that meet the energy criteria and the corresponding number of structures obtained in the MTD-PX simulation.

region	CV1	CV2	number of structures
IS	max: 0.880<br>min: 0.850	max: 0.049<br>min: 0.040	329
TS(R_1)	max: 0.480<br>min: 0.370	max: 0.065<br>min: 0.039	1759
TS(R_3)	max: 0.080<br>min: 0.056	max: 0.454<br>min: 0.408	692
proton-PX	max: 0.078<br>min: 0.054	max: 0.735<br>min: 0.699	428

Similarly, Supplementary Table 2 shows the region and the number of structures of each metastable states and transition states.

Supplementary Table 2. The rectangle regions that meet the energy criteria and the corresponding number of structures in the MTD-MX simulation.

region	CV1	CV2	number of structures
IS	max: 0.889<br>min: 0.845	max: 0.060<br>min: 0.040	621
TS(R_2)	max: 0.450<br>min: 0.390	max: 0.073<br>min: 0.042	1117
TS(R_{-3})	max: 0.075<br>min: 0.055	max: 0.503<br>min: 0.423	735
proton-MX	max: 0.070<br>min: 0.050	max: 0.740<br>min: 0.719	331

For regions TS(R_1) and TS(R_2), we performed further analysis, and Supplementary Table 3 shows the average distances of C_{me}-C_p/C_m/C_o and C_{me}-O_{me} of TS(R_1) and TS(R_2) structures in both MTD-PX and MTD-MX simulations. This further proves that the structures of the TS(R_1) and TS(R_2) are similar.

Supplementary Table 3. Average distances of C_{me}-C_p/C_m/C_o and C_{me}-O_{me} of TS(R_1) and TS(R_2) structures in both MTD-PX and MTD-MX simulations, with uncertainty (standard deviation).

TS (R_1/R_2)	C_{me}-C_p(Å)	C_{me}-C_m(Å)	C_{me}-C_o(Å)	C_{me}-O_{me}(Å)
MTD-PX	3.28±0.09	2.83±0.33	2.91±0.59	2.10±0.05
MTD-MX	3.60±0.47	3.22±0.09	3.07±0.52	2.11±0.02

In addition, Supplementary Figure 9(a) shows the structural analysis results at proton-PX basin of MTD-PX simulation, which suggests the formation of C_{me}-C_p bond in proton-PX as expected. Similarly, Supplementary Figure 9(b) confirms the bond formation between C_{me} and C_m. Through this, we confirmed the structures of FS in the two MTD simulations, and provided support for the calculation of free energy differences, e.g. reaction energy.

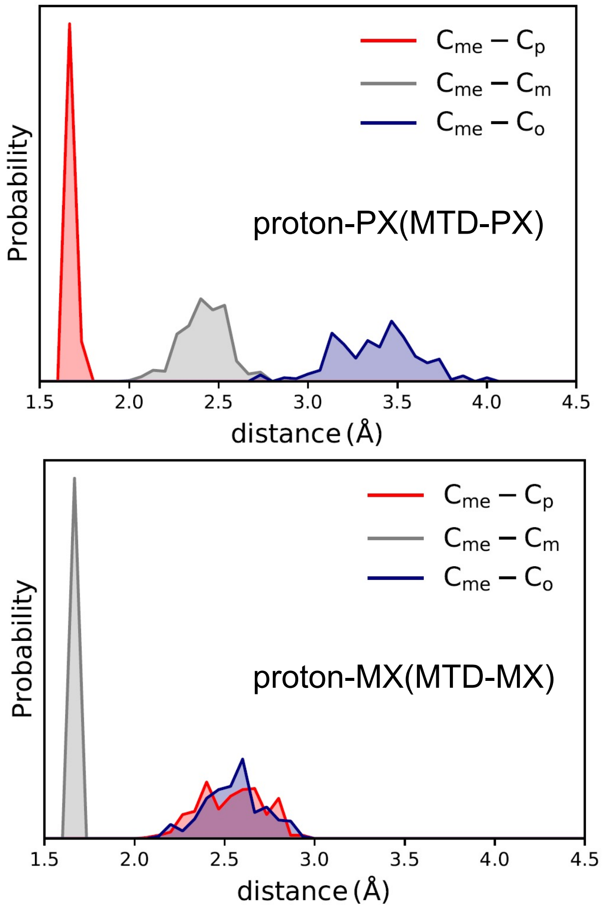

Supplementary Figure 9. The probability distribution diagrams of the bond lengths of C_{me}-C_p, C_{me}-C_m and C_{me}-C_o at proton-PX basin of MTD-PX and proton-MX basin of MTD-MX.

Supplementary Note 7. Collective variables and quadratic walls

Supplementary Table 4. Collective variables and quadratic walls applied in the MTD-PX and MTD-MX simulations.

MTD-PX

**CV**	***r₀*(Å)**	**Position**	**K^a^ (×1000 kJ mol^-1^)**	**Direction**	**hills**
CN(C_{me}-O_{me})	2.0	0.04	2625.5	Lower	Yes
CN(C_{me}-C_p)	2.0	0.03	26.255	Lower	Yes
		0.73	2625.5	Upper	
CN(O_{me}-H_{me})	1.0	0.03	262.5	Lower	No
CN(O_{me}-H_z)	1.0	0.03	262.5	Lower	No

MTD-MX

**CV**	***r₀*(Å)**	**Position**	**K^a^ (×1000 kJ mol^-1^)**	**Direction**	**hills**
CN(C_{me}-O_{me})	2.0	0.04	2625.5	Lower	Yes
CN(C_{me}-C_m)	2.0	0.03	26.255	Lower	Yes
		0.73	2625.5	Upper	
CN(O_{me}-H_{me})	1.0	0.03	262.5	Lower	No
CN(O_{me}-H_z)	1.0	0.03	262.5	Lower	No

Supplementary Note 8. Convergence tests of slow-growth simulations.

Supplementary Figure 10 and Figure 5 in the main text show the Jarzynski averaging curve obtained from five and ten, respectively, parallel tests with the error obtained from the standard deviation of work. The difference between these two simulations is rather small and within the calculated uncertainty, suggesting that ten parallel tests are sufficient.

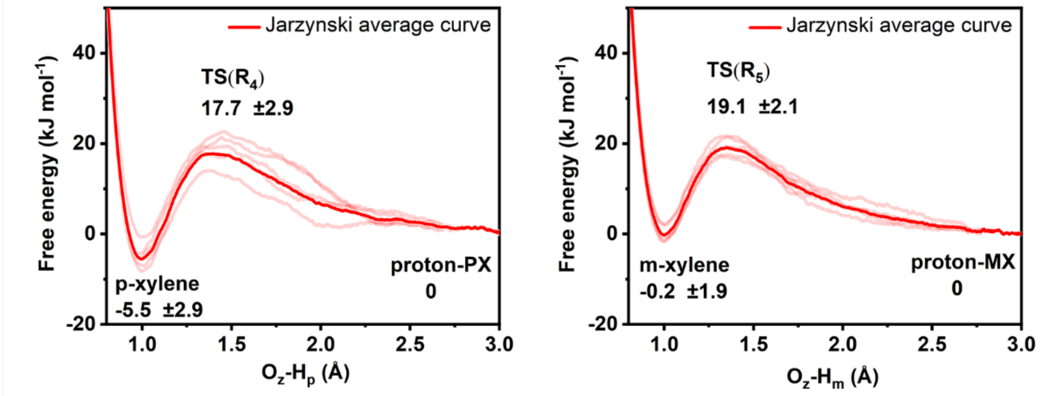

Supplementary Figure 10. Free energy profiles of proton–PX and proton–MX deprotonation reaction obtained from 5 independent trajectories. Thin lines are the work curves of individual trajectories and the thick line is the free energy profile obtained from Jarzynski equality. The values of free energy and the standard deviation of works are also shown in the figure.

To confirm that the O_z-$H_{p/m}$ distance of 3.0 Å can represent the free energy minimum, we set the reaction coordinate change rate to 0.000109 Å frame^{-1}, while the initial structure and other parameters remain unchanged. As for proton–PX, the free energy change is small and fluctuates around 0, and for proton–MX the free energy increases gradually, demonstrating that initial reaction coordinate approaches to the free energy minimum.

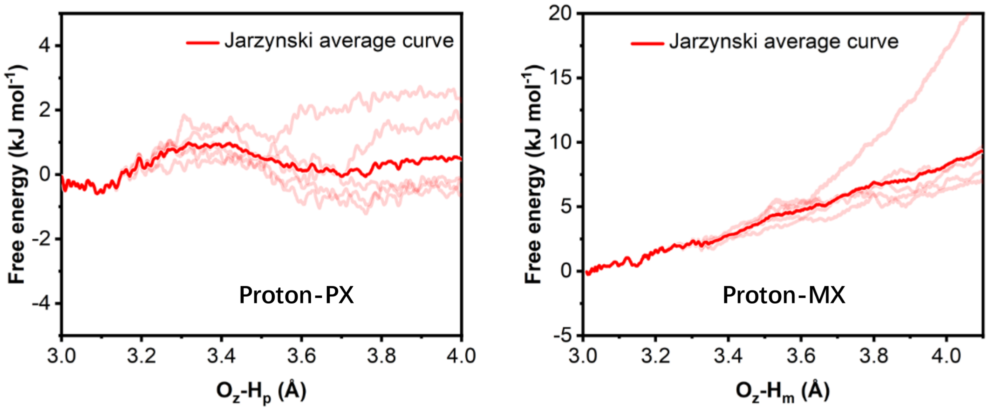

Supplementary Figure 11. Free energy profiles of proton–PX and proton–MX deprotonation reaction in the O_z-$H_{p/m}$ distance range of 3.0 to 4.0 Å.

Supplementary Note 9. Diffusion barriers

The values of diffusion barriers and its uncertainties were shown in Supplementary Table 5. The uncertainties of free energy barriers were obtained in the following way: Firstly, the standard deviation (σ_f) of constrained force was estimated using the block average method. Then the linear error propagation theory was used to calculate the uncertainty of free energy profiles by summing up the variance of work in each bin (σ_w^2) from minimum to maximum of the free energy profiles.

Supplementary Table 5. Diffusion barriers (in kJ mol^{-1}) with standard deviation of m-xylene and p-xylene along the two channels in HZSM-5.

	Straight	Zigzag
m-xylene	23.2 ± 4.2	46.8 ± 4.4
p-xylene	19.4 ± 2.7	15.9 ± 4.6

Two examples trajectories of F_c were given in Supplementary Figure 12. From the trajectories of constraint force F_c, we can see that F_c is equilibrated in the range of 1000 to 5000 steps. Therefore, in each constrained MD simulation, totally 5000 steps were performed and the last 4000 steps of the trajectory were analyzed.

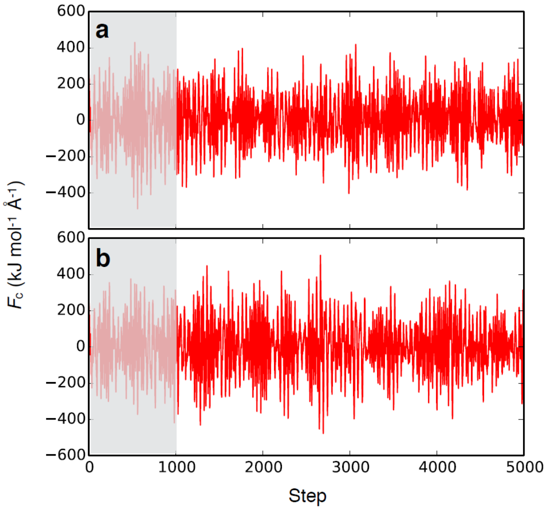

Supplementary Figure 12. The trajectories of constraint force F_c in the constrained MD simulations. Two trajectories, i.e., PX diffusion along straight channel at l = 1.0 Å (a) and 2.0 Å (b), were given as examples. The shaded regions suggest that the first 1000 steps were omitted.

Supplementary Note 10. Trajectories of CV1-CV2 in MTD-PX/MX

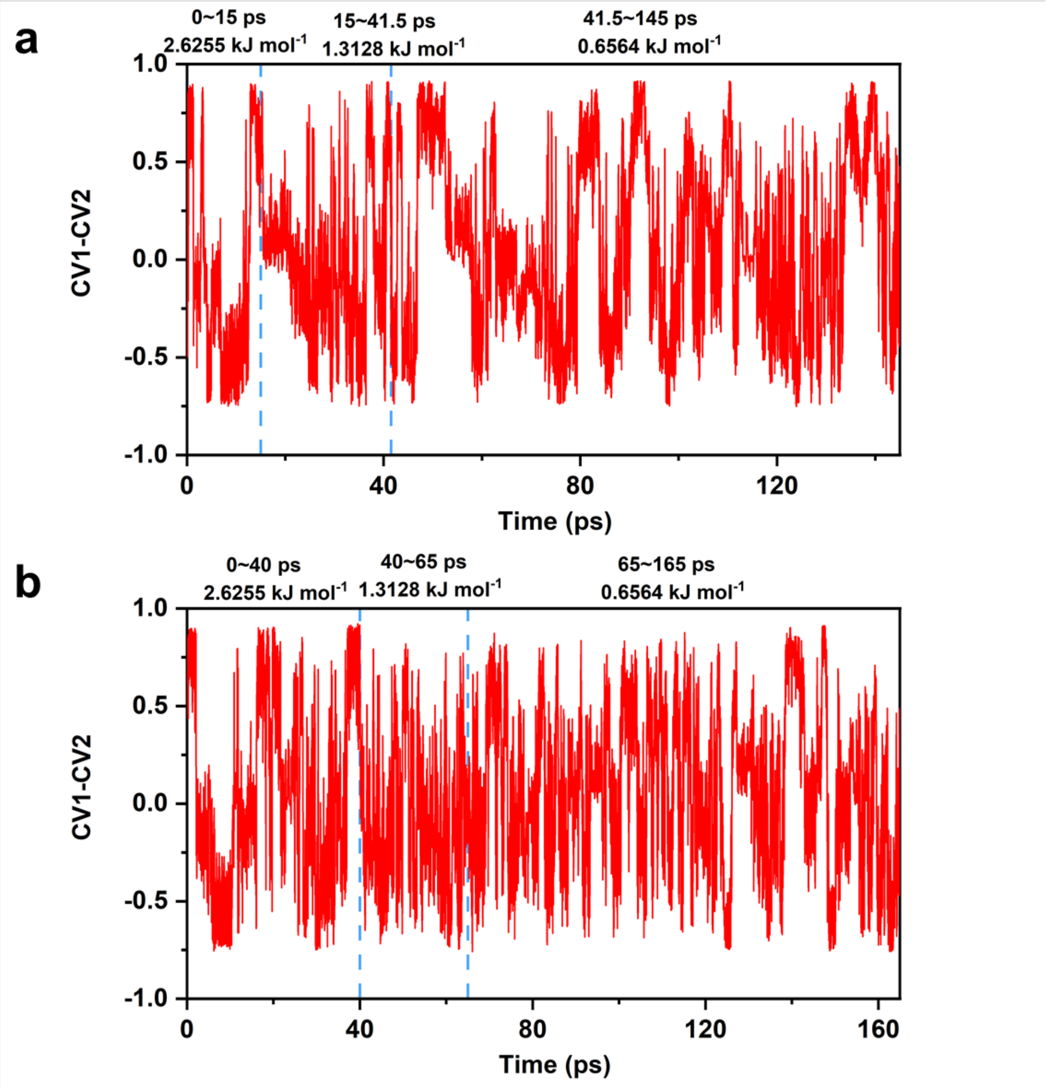

Supplementary Figure 13. The trajectories of CV1-CV2 and the moments to reduce the height of the Gaussian hill in (a) MTD-PX and (b) MTD-MX simulations.

Supplementary References

1 Moors, S. L. C., De Wispelaere, K., Van der Mynsbrugge, J., Waroquier, M. & Van Speybroeck, V. Molecular dynamics kinetic study on the zeolite-catalyzed benzene methylation in ZSM-5. *ACS Catal.* **3**, 2556-2567, doi:10.1021/cs400706e (2013).

2 Bussi, G. & Laio, A. Using metadynamics to explore complex free-energy landscapes. *Nat. Rev. Phys.* **2**, 200-212, doi:10.1038/s42254-020-0153-0 (2020).
